# Deep convolutional neural networks for multiple histologic types of ovarian tumors classification in ultrasound images

**DOI:** 10.3389/fonc.2023.1154200

**Published:** 2023-06-23

**Authors:** Meijing Wu, Guangxia Cui, Shuchang Lv, Lijiang Chen, Zongmei Tian, Min Yang, Wenpei Bai

**Affiliations:** ^1^ The Department of Gynecology and Obstetrics, Beijing Shijitan Hospital, Capital Medical University, Beijing, China; ^2^ The Department of Electronics and Information Engineering, Beihang University, Beijing, China

**Keywords:** deep convolutional neural network, deep learning, ultrasound, benign ovarian tumor, high-grade serous carcinoma

## Abstract

**Objective:**

This study aimed to evaluate and validate the performance of deep convolutional neural networks when discriminating different histologic types of ovarian tumor in ultrasound (US) images.

**Material and methods:**

Our retrospective study took 1142 US images from 328 patients from January 2019 to June 2021. Two tasks were proposed based on US images. Task 1 was to classify benign and high-grade serous carcinoma in original ovarian tumor US images, in which benign ovarian tumor was divided into six classes: mature cystic teratoma, endometriotic cyst, serous cystadenoma, granulosa-theca cell tumor, mucinous cystadenoma and simple cyst. The US images in task 2 were segmented. Deep convolutional neural networks (DCNN) were applied to classify different types of ovarian tumors in detail. We used transfer learning on six pre-trained DCNNs: VGG16, GoogleNet, ResNet34, ResNext50, DensNet121 and DensNet201. Several metrics were adopted to assess the model performance: accuracy, sensitivity, specificity, FI-score and the area under the receiver operating characteristic curve (AUC).

**Results:**

The DCNN performed better in labeled US images than in original US images. The best predictive performance came from the ResNext50 model. The model had an overall accuracy of 0.952 for in directly classifying the seven histologic types of ovarian tumors. It achieved a sensitivity of 90% and a specificity of 99.2% for high-grade serous carcinoma, and a sensitivity of over 90% and a specificity of over 95% in most benign pathological categories.

**Conclusion:**

DCNN is a promising technique for classifying different histologic types of ovarian tumors in US images, and provide valuable computer-aided information.

## 1 Introduction

Ovaries are an important part of the female reproductive system. Ovarian tumors have many different histological types. Over 80% of patients with ovarian tumors are benign lesions. The histopathological types of benign ovarian tumors are mainly concentrated in serous cystadenoma, endometriotic cyst, mature cystic teratoma and mucinous cystadenoma, accounting for nearly 90% of patients operated for ovarian tumors (1). The treatments vary from one to another according to the histologic types and biological behavior of ovarian tumors, patient’s age and fertility needs. The surgeon needs to figure out the tumor size, benign or malignant, histologic type as clear as possible to make treatment decision (2). It is of vital important to figure out the histological types of ovarian tumors before surgery.

The preoperative diagnosis of ovarian tumors is highly dependent on imaging tests. Ultrasonography is widely used in the clinical diagnosis of ovarian tumors because of its simplicity, non-invasiveness, non-radiation, safety and affordability (3–5). Ultrasound images are interpreted manually by sonographers, and their accuracy plays an important role in the diagnosis and assessment of disease. However, variability in diagnostic results is inevitable due to professional knowledge, clinical experience, physiological fatigue, and subjective differences (6). With the development of artificial intelligence technology, the combination of computer technology and medical image analysis will be expected to provide new solutions in terms of cost, efficiency, and accuracy.

In recent years, deep learning analysis has been widely used in medical image processing (7). Scientists have proposed many methods to process medical images using deep neural network models, such as Convolutional Neural Networks (CNN) (8), Fully Convolutional Networks (FCN) (9), or Recurrent Neural Network (RNN) (10), which can segment and assisting diagnose diseases of medical images. There are researches in brain (11), lung (12), breast (13), liver (14), vascular artery (15), thyroid (16) and more. The DCNN can assist in diagnosing diseases by pre-processing images through algorithms.

However, research and application of deep learning-based US image analysis in the field of ovarian tumors is rare. Current researches focus on the benign and malignant classifications (17–21), but the treatment is closely related to the specific pathological categories. The subdivided pathological categories can be helpful to the physician in making a surgical decision. In this project, based on our ovarian ultrasound images, we propose to build a deep learning-based prediction model of ovarian tumors, which can classify the pathological categories in detail, and evaluate its clinical value in assisting the diagnosis of ovarian tumors, using postoperative histopathological examination results as confirmation criteria.

## 2 Material and methods

This retrospective study was approved by the institutional review board of Beijing Shijitan Hospital, and written informed consent was waived (approval number sjtky11-1x-2022 (085)).

### 2.1 Dataset collection

This study retrospectively collected patients with benign and malignant ovarian tumors who underwent surgery for ovarian tumors at Beijing Shijitan Hospital from January 2019 to June 2021 and underwent preoperative ultrasound, and were confirmed by postoperative histopathological examination.

Inclusion criteria were as follows: patients who had preoperative diagnostic-quality US showing at least one persistent ovarian tumor (excluding physiologic cysts), and underwent surgery with subsequent histopathologic results.

**Figure 1:**
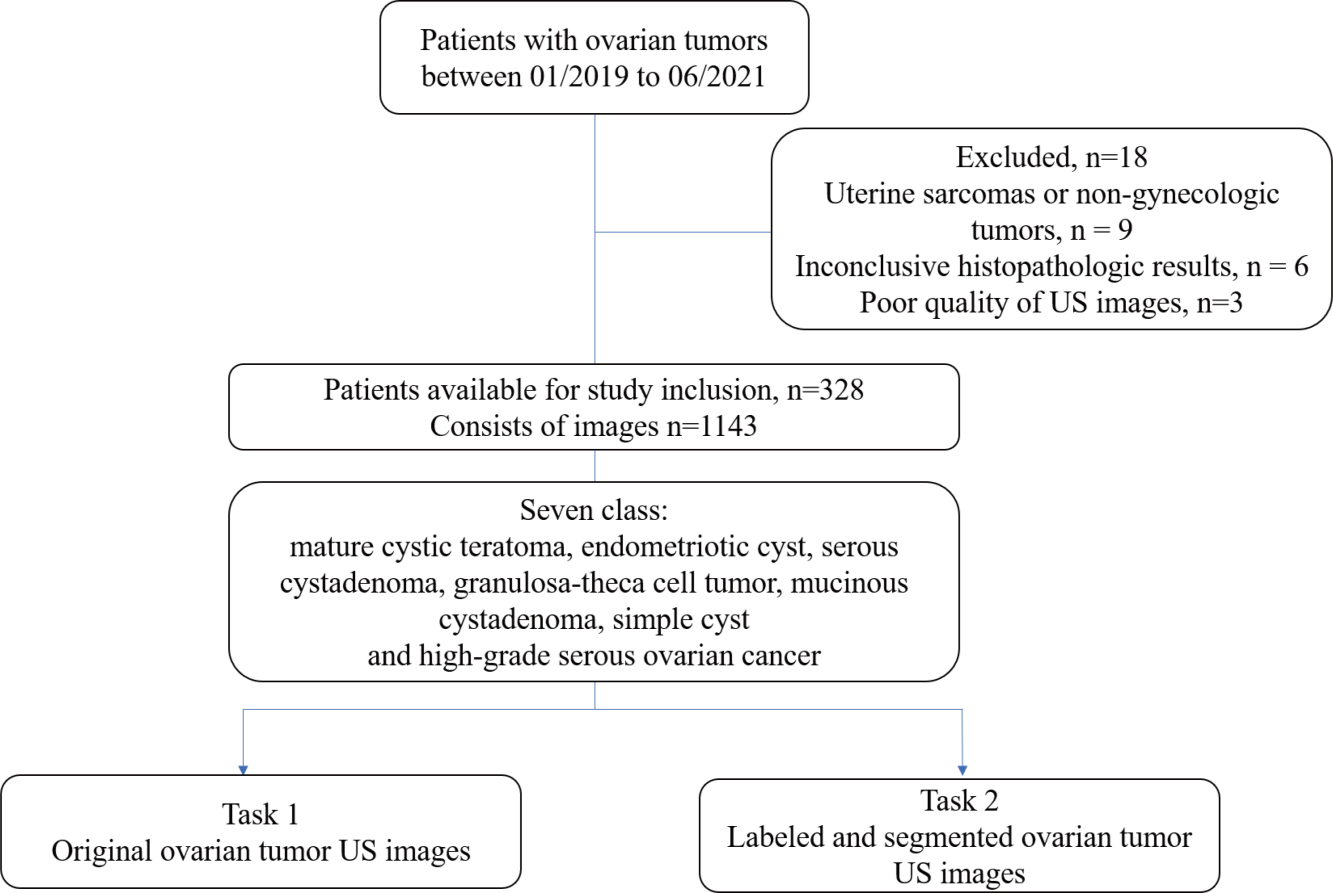
Flowchart of the experimental design and patient recruitment.

Exclusion criteria were histopathologically confirmed uterine sarcoma or non-gynecologic tumors, inconclusive histopathologic results, or US images of ovarian tumor showed blurred boundary, and the boundary of the lesions could not be delineated. The patient flowchart is shown in **Figure 1**.

### 2.2 Data preprocessing

After sonographer training, lesions were delineated along macroscopic lesion borders or anatomical structures using labelme software. The delineation results of the lesions were reviewed by the sonographer with more than five years of experience. Given the limitations of medical image acquisition, we applied data augmentation techniques to image processing to reduce overfitting and improve generalization. Data enhancing technology was used to randomly crop and flip the original images. Gray features and texture information of the lesion cannot be altered during this process. All augmented images were resized to 256 * 256 pixels. Python (version 3.7.3) was used to conduct all processing steps.

### 2.3 DCNN model training and interpretation

**Figure 2:**
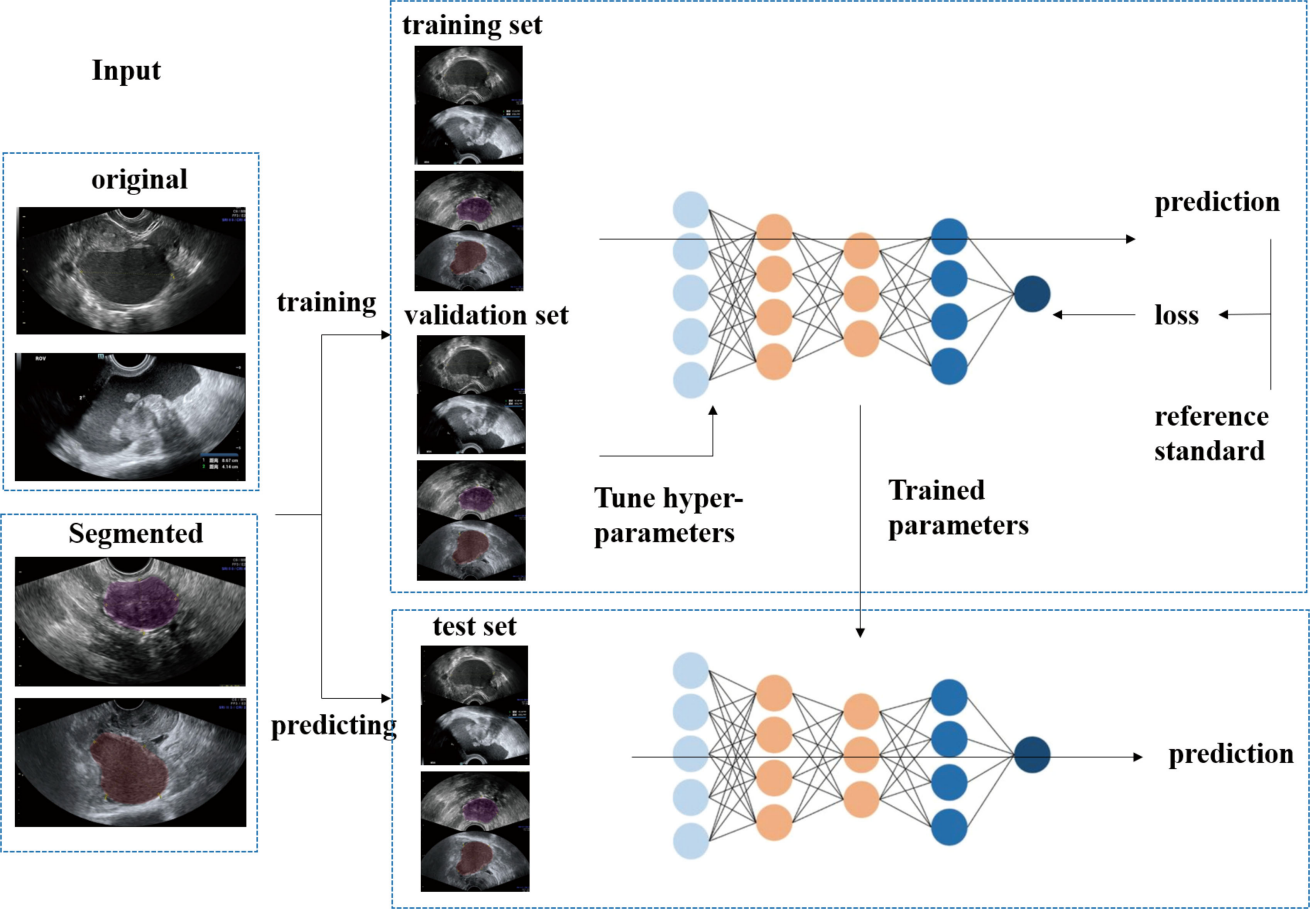
The flow diagram shows the image input and the main processing steps for algorithms based on deep learning.

The procedure used for image processing is shown in **Figure 2**. Six representative DCNN architectures, VGG16, GoogleNet, ResNet34, ResNext50, DensNet121 and DensNet201 (22–26), were used to identify different histological types of ovarian tumors based on US images. We classify US images into 7 classes: the benign cohort was divided into six categories: mature cystic teratoma, endometriotic cyst, serous cystadenoma, granulosa theca cell tumor, mucinous cystadenoma, and simple cyst, and one malignant category, high-grade serous ovarian cancer. Each subgroup was sampled at 70%, 10%, and 20% and pooled together to construct training, validation, and test data sets. They were all trained and validated by the DCNN.

During training, the weights of the neural network were updated using the Adam optimizer with an initial learning rate of 0.001 and a batch size of 16. All models were trained for 200 epochs; the momentum is set to 0.9. An Intel(R) Xeon(R) E5-2690 v4 CPU and Nvidia GeForce GTX 1080 GPU were used for models training. For classification, the transfer learning methods of these networks were also used (27, 28). We then used back-propagation to fine tune the parameters of the fully connected layer of the network on our dataset. All programs were executed in Python version 3.7.3.

We used the Classifier Activator Map (CAM) method to visualize the important regions leading to the decision of the deep learning model in order to improve the interpretability of our model (29). We obtained heat maps explaining which parts of an input US image were focused by the DCNN. All heat maps were generated using the OpenCV package (version 4.3.0.36).

### 2.4 Statistical analysis

Our study investigated different DCNN models, obtained experimental results, and calculated indicators based on them. The area under the receiver operating characteristic curve, accuracy, sensitivity, specificity, and F1 score were used to evaluate the performance of the 7-class classification task. Differences between models were considered statistically significant when P < 0.05. These measures were calculated using the NumPy (version 1.16.2).

## 3 Results

### 3.1 Patient and US images characteristics

A total of 328 patients with ovarian tumors (290 benign ovarian tumors, 38 high-grade serous carcinomas), were enrolled in this study retrospectively. There were a total of 1142 individual US images. And they were further divided into different pathological categories. The characteristics of patients and image numbers of different pathological categories are shown in **Table 1**.

**Table 1:** Characteristics of patients and image numbers of different pathological categories in the Training, Validation, and Test Sets.

Category		Patients’ characteristics	Images	
			Training and Validation sets	Test sets
Total number		328	914	228
Age (y)	Median Range	56 (18–85)	55 (18–84)	56 (20–85)
Histological types	Class group			
Endometriotic cysts	Class 1	80 (24.4)	231 (25.3)	58 (25.3)
Serous cystadenomas	Class 2	66 (20.1)	173 (18.9)	43 (18.8)
Mature cystic teratoma	Class 3	92 (28.0)	268 (29.4)	67 (29.3)
Granulosa-theca cell tumor	Class 4	27 (8.2)	71 (7.8)	18 (7.9)
Simple cyst	Class 5	21 (6.4)	48 (5.3)	12 (5.2)
Mucinous cystadenoma	Class 6	25 (7.6)	80 (8.8)	20 (8.7)
High-grade serous carcinoma	Class 7	17 (5.2)	42 (4.7)	11 (4.4)
Maximum lesion diameter (mm)*		57.0 (41.3–80.6)	58.0 (40.0–80.1)	56.0 (39.0–81.0)

Data in parentheses are percentages. *Data are medians; data in parentheses are IQRs.

### 3.2 Performance of the seven-class classification task

On the original US image test sets, the performance of DCNN models with transfer learning is shown in **Table 2**. The ResNext50 model achieved a higher accuracy of 0.811 in the test set with an AUC of 0.95 (95% CI: 0.931, 0.969), a sensitivity of 70.2% and a specificity of 96.7%.

**Table 2:** Diagnostic performance of six Deep convolutional neural networks (DCNN) models for classification of multiple histologic types of ovarian tumors in the original ultrasound image test set.

	VGG16	GoogleNet	ResNet34	ResNext50	DensNet121	DensNet201
SEN	0.679 [0.638,0.72]	0.563 [0.52,0.606]	0.695 [0.655,0.735]	0.702 [0.662,0.742]	0.576 [0.533,0.619]	0.702 [0.662,0.742]
SPEC	0.958 [0.94,0.976]	0.943 [0.923,0.963]	0.961 [0.944,0.978]	0.967 [0.951,0.983]	0.947 [0.927,0.967]	0.96 [0.943,0.977]
ACC	0.768 [0.731,0.805]	0.671 [0.63,0.712]	0.763 [0.726,0.8]	0.811 [0.777,0.845]	0.693 [0.653,0.733]	0.768 [0.731,0.805]
AUC	0.95 [0.931,0.969]	0.87 [0.841,0.899]	0.95 [0.931,0.969]	0.95 [0.931,0.969]	0.92 [0.896,0.944]	0.96 [0.943,0.977]
PPV	0.715 [0.675,0.755]	0.607 [0.564,0.65]	0.645 [0.603,0.687]	0.708 [0.668,0.748]	0.586 [0.543,0.629]	0.734 [0.695,0.773]
NPV	0.959 [0.942,0.976]	0.944 [0.924,0.964]	0.958 [0.94,0.976]	0.967 [0.951,0.983]	0.948 [0.929,0.967]	0.959 [0.942,0.976]
F1-Score	0.693 [0.653,0.733]	0.571 [0.528,0.614]	0.662 [0.621,0.703]	0.7 [0.66,0.74]	0.578 [0.535,0.621]	0.711 [0.671,0.751]

Data in brackets are 95% CIs. ACC, accuracy; SEN, sensitivity; SPEC, specificity; AUC, area under the receiver operating characteristic curve; NPV, negative predictive value; PPV, positive predictive value.

**Figure 3:**
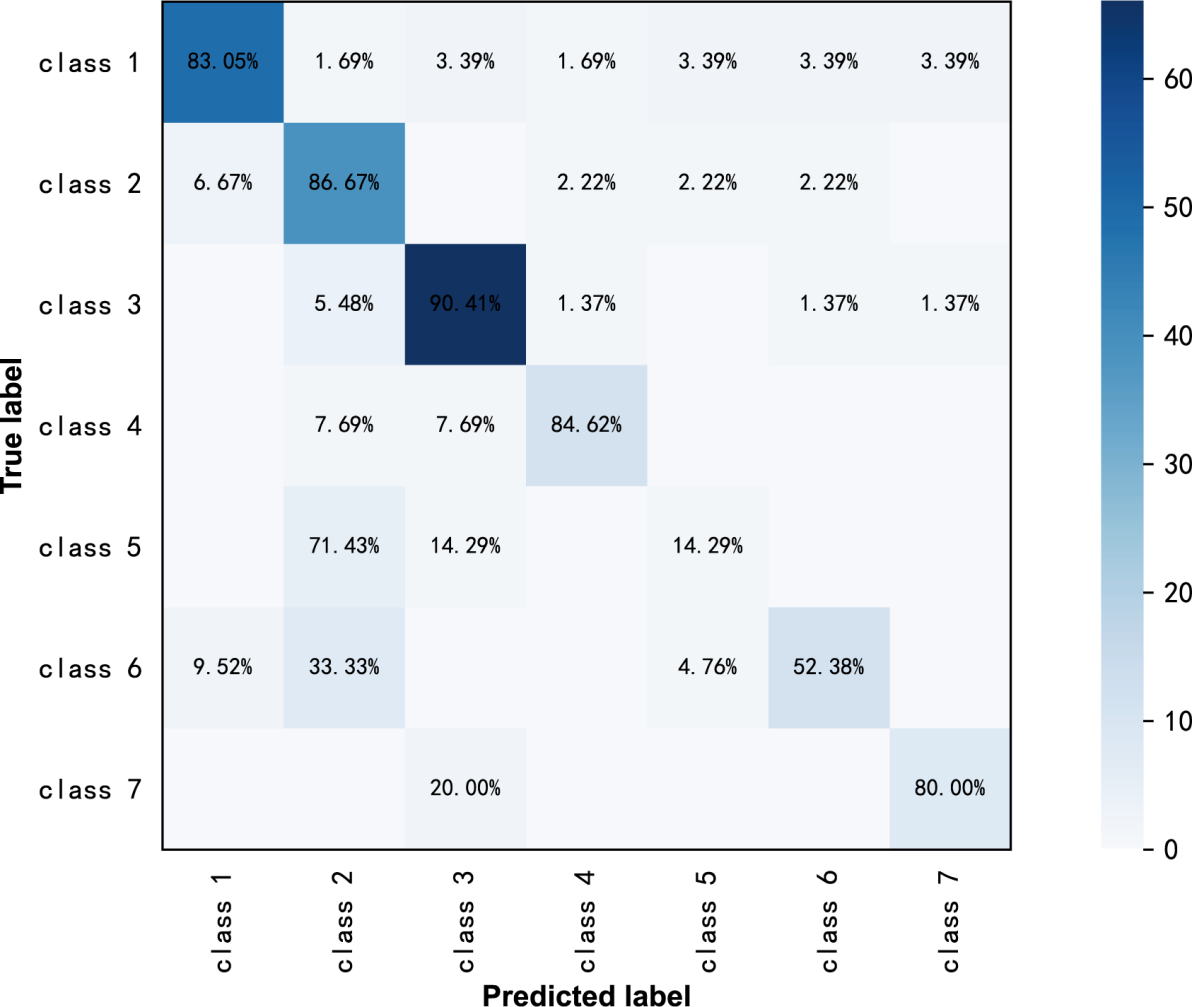
Confusion matrix of ResNext50 models for seven-class classification in original ultrasound images.

ResNext50 achieved a sensitivity of 80.00% - 90.41% in most of the subdivided pathological categories. In high-grade serous carcinoma (class 7), the sensitivity was 80%, the specificity was 98.6% and the F1 score was 0.762 (**Table 3**). The confusion matrix is shown in **Figure 3**.

**Table 3:** Diagnostic Performances of ResNext50 models seven-class classification in original ultrasound images.

	SEN	SPEC	F1-score
Endometriotic cysts	0.831 [0.798,0.864]	0.97 [0.955,0.985]	0.867 [0.837,0.897]
Serous cystadenomas	0.867 [0.837,0.897]	0.902 [0.876,0.928]	0.765 [0.728,0.802]
Mature cystic teratoma	0.904 [0.878,0.93]	0.961 [0.944,0.978]	0.91 [0.885,0.935]
Granulosa-theca cell tumor	0.846 [0.814,0.878]	0.986 [0.976,0.996]	0.815 [0.781,0.849]
Simple cyst	0.143 [0.112,0.174]	0.982 [0.97,0.994]	0.167 [0.134,0.2]
Mucinous cystadenoma	0.524 [0.48,0.568]	0.981 [0.969,0.993]	0.611 [0.568,0.654]
High-grade serous carcinoma	0.8 [0.765,0.835]	0.986 [0.976,0.996]	0.762 [0.725,0.799]

**Figure 4:**
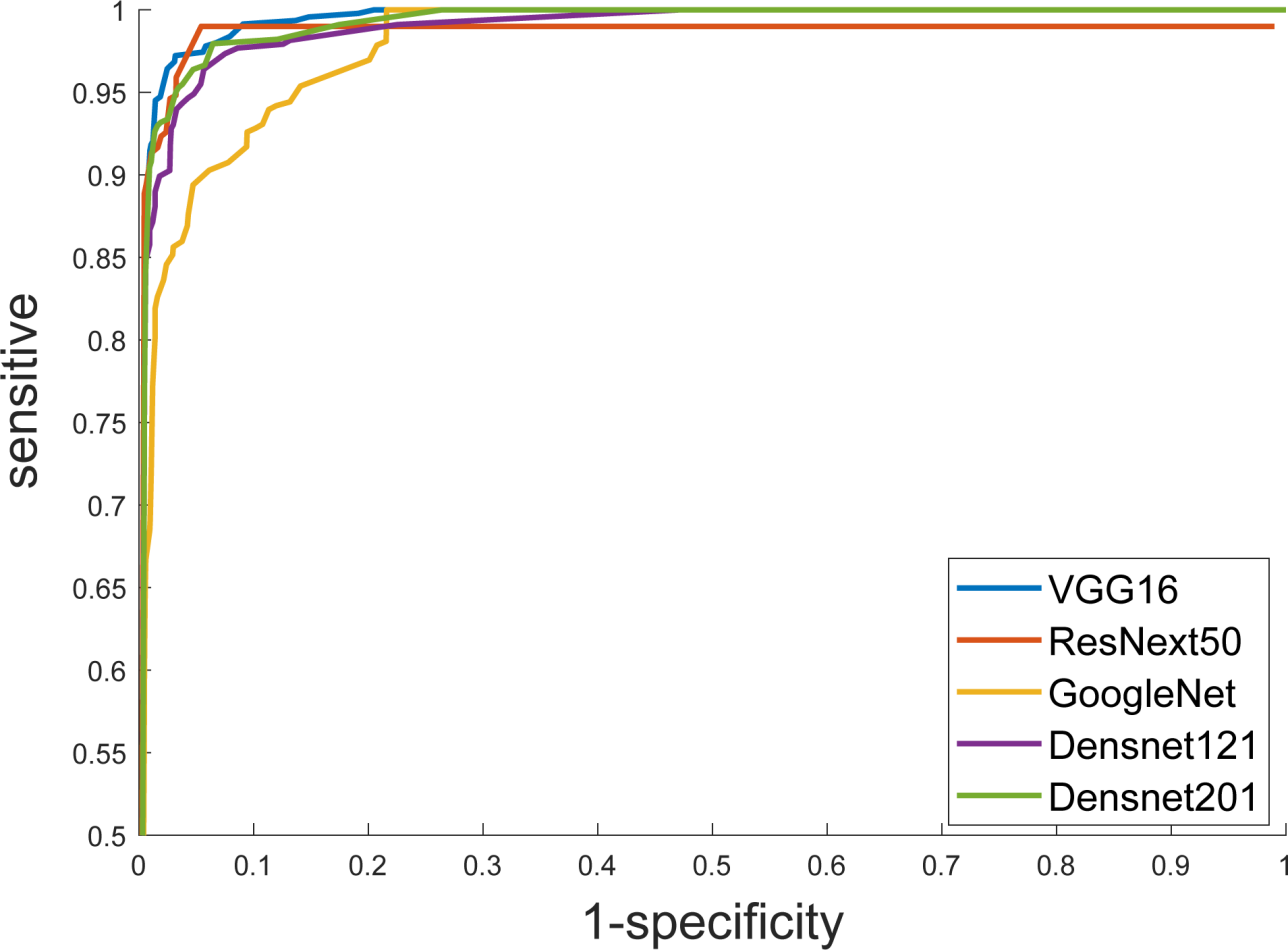
Receiver operating characteristic (ROC) curves show the diagnostic performance of six DCNN models.

Furthermore, we put the masked US images into different DCNN models, and obtained better diagnostic results than that of the original US images. The ResNext50 model using transfer learning methods also showed the best discrimination performance, with an AUC of 0.997 (**Figure 4**), a sensitivity of 89.5%, a specificity of 99.2% and F1 score of 0.905 (**Table 4**). The AUC in each model was not significantly different from the other, as p-value > 0.05 in each comparison.

**Table 4:** Diagnostic performances of six deep convolutional neural network models for classifying multiple histologic types of ovarian tumors in the in the labeled ultrasound images test set.

	VGG16	GoogleNet	ResNet34	ResNext50	DensNet121	DensNet201
SEN	0.854 [0.823,0.885]	0.787 [0.751,0.823]	0.814 [0.78,0.848]	0.895 [0.868,0.922]	0.862 [0.832,0.892]	0.876 [0.847,0.905]
SPEC	0.982 [0.97,0.994]	0.974 [0.96,0.988]	0.985 [0.974,0.996]	0.992 [0.984,1]	0.982 [0.97,0.994]	0.988 [0.978,0.998]
ACC	0.899 [0.873,0.925]	0.855 [0.824,0.886]	0.912 [0.887,0.937]	0.952 [0.933,0.971]	0.895 [0.868,0.922]	0.934 [0.912,0.956]
AUC	0.99 [0.981,0.999]	0.98 [0.968,0.992]	0.99 [0.981,0.999]	0.997 [0.992,1]	0.991 [0.983,0.999]	0.995 [0.989,1]
PPV	0.863 [0.833,0.893]	0.824 [0.791,0.857]	0.804 [0.769,0.839]	0.918 [0.894,0.942]	0.866 [0.836,0.896]	0.924 [0.901,0.947]
NPV	0.983 [0.972,0.994]	0.976 [0.963,0.989]	0.985 [0.974,0.996]	0.992 [0.984,1]	0.982 [0.97,0.994]	0.989 [0.98,0.998]
FI-Score	0.856 [0.825,0.887]	0.803 [0.768,0.838]	0.81 [0.776,0.844]	0.905 [0.879,0.931]	0.856 [0.825,0.887]	0.895 [0.868,0.922]

**Figure 5:**
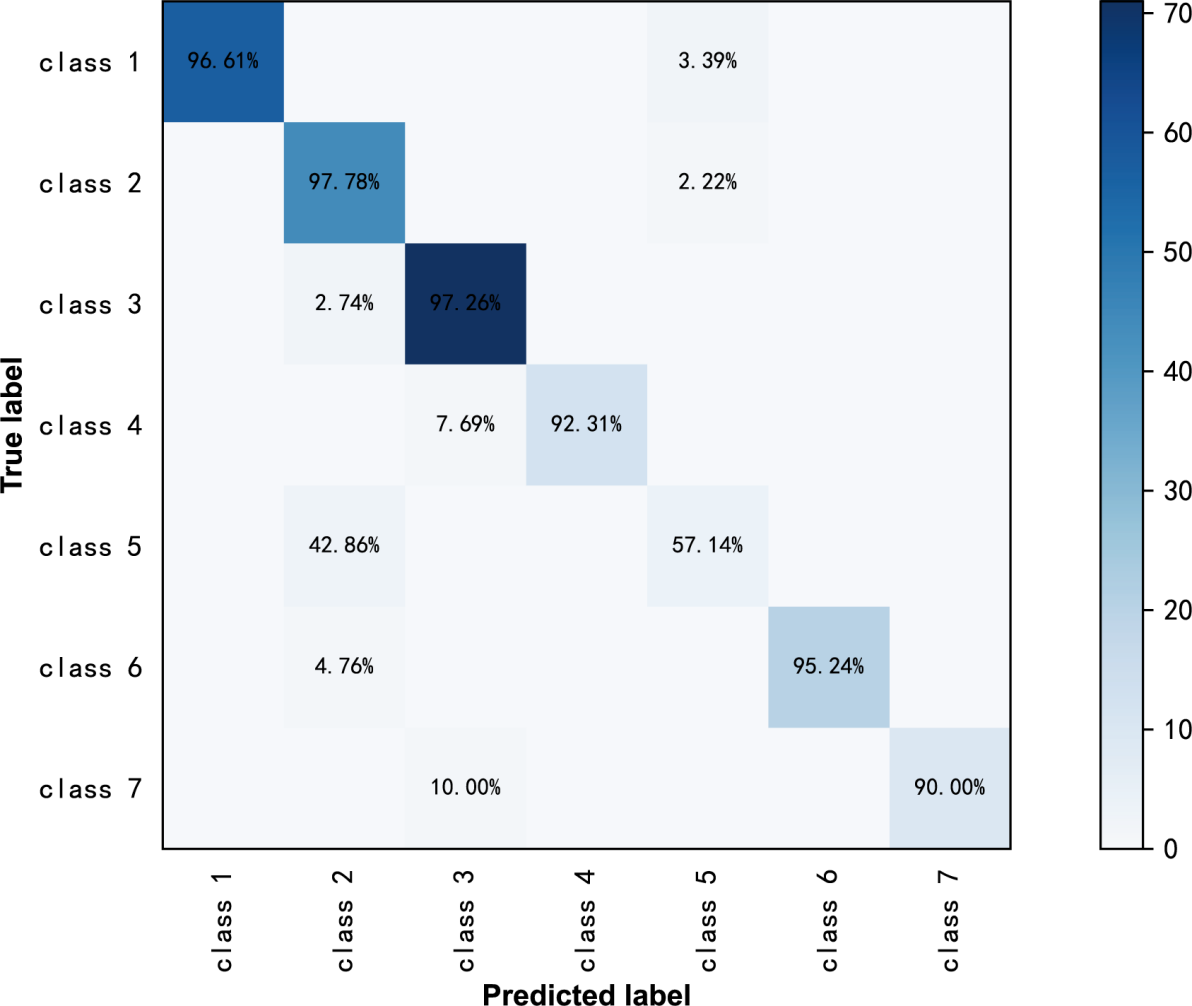
Confusion matrix of ResNext50 models for seven-class classification in labeled ultrasound images.

ResNext50 achieved sensitivity greater than 90% in subdivided pathologic categories. The sensitivity was 90% and the specificity was 99.2%, and the F1-Score was 0.947 in the class high-grade serous carcinoma (**Table 5**). The confusion matrix is shown in **Figure 5**.

**Table 5:** Diagnostic Performance of ResNext50 models seven-class classification in labeled ultrasound images.

	SEN	SPEC	F1-score
Endometriotic cysts	0.966 [0.95,0.982]	0.995 [0.989,1]	0.983 [0.972,0.994]
Serous cystadenomas	0.977 [0.964,0.99]	0.967 [0.951,0.983]	0.926 [0.903,0.949]
Mature cystic teratoma	0.972 [0.958,0.986]	0.987 [0.977,0.997]	0.973 [0.959,0.987]
Granulosa-theca cell tumor	0.923 [0.9,0.946]	0.993 [0.986,1]	0.96 [0.943,0.977]
Simple cyst	0.571 [0.528,0.614]	0.986 [0.976,0.996]	0.571 [0.528,0.614]
Mucinous cystadenoma	0.952 [0.933,0.971]	0.99 [0.981,0.999]	0.976 [0.963,0.989]
High-grade serous carcinoma	0.9 [0.874,0.926]	0.992 [0.984,1]	0.947 [0.927,0.967]

### 3.3 Visualizing and understanding DCNN

**Figure 6:**
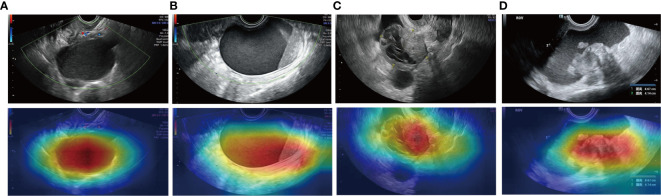
Examples of the mapping of class activation using the ResNext50 model. The model identified endometriotic cyst **(A)**, mucinous cystadenoma **(B)**, and high-grade serous ovarian cancer **(C, D)** correctly.

CAM heatmaps (**Figure 6**) show that a map of such a localization is completely generated by the fully trained ResNext50 model without any manual annotation. The red and yellow area represents an area which has the greatest predictive significance in the ResNext50 model; the blue and green backgrounds reflect areas with weaker predictive values. Redder feature colors indicate a higher DCNN score. For the images that were correctly diagnosed, the DCNN focused on the same areas as the clinicians did.

## 4 Discussion

Diagnosing the pathological categories of ovarian tumors from US images can help physicians select more appropriate treatments. In this study, we used six DCNNs: VGG16, GoogleNet, ResNet34, ResNext50, DensNet121, and DensNet201 on ovarian tumor US images and set up 7 classification tasks in which benign ovarian lesions were divided into mature cystic teratoma, endometriotic cyst, serous cystadenoma, granulosa theca cell tumor, mucinous cystadenoma, and simple cyst. The DCNN model was evaluated on the original and labeled ovarian tumor US images. The results showed that ResNext50 had the highest overall accuracy. And better results were obtained in the segmented ultrasound images without noise interference.

In recent years, medical image analysis based on convolutional neural networks has been widely applied to computer-aided diagnosis of diseases. Resnet achieved the highest classification accuracy in the 2015 ImageNet competition. In ovarian tumor ultrasound images, previously developed models have mostly been used to discriminate between benign and malignant ovarian lesions. There has been no study on the classification of ovarian tumors into different pathological categories, which is important for the selection of treatment and surgical procedure. We found that ResNext50 achieved better overall accuracy in the test set with an area under the receiver operating characteristic curve (AUC) of 0.997 (95% CI: 0.992,1), sensitivity of 89.5% and specificity of 99.2%. Among them, the sensitivity (90% *vs* 96%), specificity (99.2% *vs* 87%), and AUC (0.99 *vs* 0.97) for malignant ovarian tumor reached a similar result as the expert assessment by Chen et al. (18).

From the confusion matrix in **Figure 4**, we can see that the high-grade serous carcinoma has a 10% chance of being misclassified as a serous cystadenoma, which is likely to occur in the early stages of the carcinoma. In this condition, further radiographic evaluation is needed, such as contrast-enhanced ultrasound. Simple cysts can be easily classified as serous cystadenomas because of the similarities in the ultrasound appearance of the two lesions. Endometriotic cyst, mature cystic teratoma, granulosa theca cell tumor and mucinous cystadenoma can be correctly diagnosed with more than 95% accuracy, demonstrating the excellent performance of the ResNext50 model.

A heatmap of CAM output was also provided in **Figure 6**. Most ovarian tumors are cystic solid tumors. The degree of malignancy increases with the number of solid components present in the ultrasound images. **Figure 6** shows that DCNN focuses on the extent of the lesion and even the solid component of the tumor.

In this study, images were manually cropped. The DCNN produced better diagnostic results on the labeled US images than on the original US images. It is important to help the computer locate the lesion in advance. Despite the fact that most medical deep learning studies use manual ROI selection (30), the potential benefit of auto-segmentation should be explored in future studies.

Our study has several limitations. First, our study is a single-center study and a multi-center evaluation is needed to further develop and validate our model. Second, this is a retrospective study, and the data set is limited. Prospective studies are necessary to make further improvements. Third, our study is based on single-modal US images to trained the DCNN models. Multiple US images are used in clinical practice to diagnose ovarian cancer, including gray scale, color Doppler, and power Doppler. Investigating the performance of DCNN in multiple US images could be a next step.

It is important to emphasize that computer-assisted image analysis should only be used as an aid in the triage of patients and should not be used to make a definitive diagnosis. Nevertheless, we demonstrate that deep learning algorithms based on ultrasound images can predict the type of ovarian tumors, which have the potential to be clinically useful in the triage of women with an ovarian tumor.

## 5 Conclusions

In conclusion, we demonstrated that DCNNs can achieve high accuracy in distinguishing multiple pathological categories from ovarian ultrasound images. The DCNN-based model may prove to be a powerful clinical decision-support tool with more samples and further model calibration.

## Data availability statement

The original contributions presented in the study are included in the article/supplementary material. Further inquiries can be directed to the corresponding author.

## Author contributions

WB, MY, and MW: study design and methods development. GC, SL, and ZT: lesions identification and marking. MW, SL and LC: results inference and implementation of methods. MW, GC, and SL: manuscript writing. WB, MY, MW, GC, SL, and LC: manuscript/results proof read and approval. All authors contributed to the article and approved the submitted version.
